# Effects of perinatal oxycodone exposure on the cardiovascular response to acute stress in male rats at weaning and in young adulthood

**DOI:** 10.3389/fphys.2013.00085

**Published:** 2013-04-24

**Authors:** Thitinart Sithisarn, Henrietta S. Bada, Richard J. Charnigo, Sandra J. Legan, David C. Randall

**Affiliations:** ^1^Department of Pediatrics, College of Medicine, University of KentuckyLexington, KY, USA; ^2^Department of Biostatistics, College of Public Health and Department of Statistics, College of Arts and Sciences, University of KentuckyLexington, KY, USA; ^3^Department of Physiology, College of Medicine, University of KentuckyLexington, KY, USA

**Keywords:** oxycodone, opiate, blood pressure, sympathetic, conditioning, classical

## Abstract

Oxycodone (OXY) is one of the most commonly abused opiates during pregnancy. Perinatal opiate exposure (POE) is associated with neurobehavioral and hormone changes. Little is known about the effects of perinatal OXY on the cardiovascular (CV) responses to stress.

**Objectives:** to determine the effects of POE on: (1) CV responses to acute stress and ability to discriminate using a classical conditioning paradigm; (2) changes in CV response to the paradigm and retention of the ability to discriminate from postnatal day (PD) 40 to young adulthood.

**Methods:** Pregnant rats were given i.v. OXY or vehicle (CON) daily. OXY and CON males were fitted with BP telemetry units. Offspring were classically conditioned by following a pulsed tone (CS+) with tail shock. A steady tone (CS−) was not followed by shock. BP and HR were recorded during resting periods and conditioning. Changes in BP, HR from composite analysis were compared. The paradigm was repeated on PD 75.

**Results:** At PD 40, OXY rats had a lower baseline mean BP (OXY: 114.8 ± 1.0 vs. CON: 118.3 ± 1.0 mm Hg; mean ± SEM) but larger amplitude of the conditional BP increase during the stress response (OXY: +3.9 ± 0.4 vs. CON: +1.7 ± 0.4 mm Hg). Both OXY and CON rats were able to discriminate between CS+ and CS−. At PD 75, the effects of OXY on the increased amplitude of the conditional BP had dissipated (CON: +3.4 ± 2.3 vs. OXY: +4.5 ± 1.4 mm Hg). BP responses to the stress and non-stress stimuli did not differ in the OXY group, suggesting that OXY may have decreased the ability of the offspring to discriminate (OXY: CS+: 147.1 ± 1.6, CS−: 145.9 ± 1.6 mm Hg vs. CON: CS+: 155.4 ± 2.7, CS−: 147.8 ± 2.7 mm Hg).

**Conclusion:** POE is associated with subtle alterations in stress CV responses in weanling rats which dissipate when the conditioning is repeated at an early adult age. Although POE effect on the ability to discriminate at weanling age could not be detected, POE may impair retention of this ability in adulthood.

## Introduction

Opiate dependence during pregnancy continues to be a major public health problem. Although the rate of illicit drug use among pregnant women aged 15–44 years remained unchanged at 4.5% based on data averaged for 2008 and 2009, the rate of current illicit drug use among women aged 15–44 who were not pregnant continued to rise to 10.6% in 2008 to 2009 reported by National Survey on Drug Use and Health (NSDUH) (SAMHSA and NHSDA, 2008, 2009, 2010, 2011). Oxycodone (OXY), a relatively new and powerful opiate analgesic, is widely abused by pregnant women, and has become one of the most popular illicit drugs second only to marijuana. An epidemiologic study specifically reporting the prevalence of OXY use during pregnancy is still lacking, but NSDUH reported as high as 5.9 million or 2.3% of the United State population aged 12 or older as lifetime OXY users (SAMHSA and NHSDA, 2008, 2009, 2010, 2011). OXY displays a significant affinity to the kappa (κ)-opioid receptor (OR) (Ross and Smith, 1997) with a relatively low affinity to Mu (μ)-ORs compared to morphine (Chen et al., 1991). OXY crosses the blood-brain barrier rapidly; thus brain concentrations are three times higher than those in blood (Bostrom et al., 2006). Therefore, the effects of perinatal OXY exposure on the developing fetus can be quite different from those of morphine.

A number of studies have reported the suppressive effects of exposure to prenatal morphine on the stress axis and behavior. For example, prenatal morphine exposure is associated with adrenal atrophy and adrenal hypoactivity in neonatal rats (Lesage et al., 1996), decreased elevation of adrenocorticotropin (ACTH) and corticosterone (CORT), depression-like behavior during forced swim test in adult male rats (Klausz et al., 2011), and suppressed response of ACTH to a restraint stress test (RST) in adult male and female rats (Slamberova et al., 2004). However, there have been very few studies that directly examine the effects of perinatal OXY exposure on the stress axis. We have previously reported that perinatal OXY increases the pituitary (ACTH) response to a pharmacological challenge, corticotrophin releasing hormone, only in late adolescent male, but not female rat offspring (Sithisarn et al., 2008). Not only the HPA axis but also the sympathetic-adrenal-medullary (SAM) axis intimately regulates the stress response (Carrasco and Van De Kar, 2003; De Kloet et al., 2005). Blood pressure (BP) is a major cardiovascular (CV) output of the SAM axis that has never been adequately studied in the context of perinatal drug exposure. Therefore in the present study, we tested the hypothesis that perinatal OXY exposure enhances the BP response to classical aversive conditioning and impairs the offspring's ability to differentiate between stress vs. non-stress stimulus. To this end, we investigated the CV response to acute behavioral stress in the male offspring of dams exposed to OXY during pregnancy using a classical conditioning paradigm that has been previously described (Randall et al., 1994).

We chose the conditioning paradigm in part because the two “components” of the arterial BP changes during the conditioning paradigm are mediated by different underlying neuronal processes (Randall et al., 1994). The first component, or C_1_, is a transient but relatively large increase in arterial BP that ultimately derives from an intrinsic orienting response; as such, it is not “learned,” though it is modified by continued exposure to the conditional stimulus (El-Wazir et al., 2005). The second component, or C_2_ pressor event, is small, but more sustained than the C_1_ component; C_2_ must be acquired as the rats learn the association between the pulsed tone and the shock (El-Wazir et al., 2005). We compared these components of the conditional response, and associated changes in heart rate (HR), in postnatal day (PD) 40 offspring of dams exposed to OXY or vehicle during gestation. We used an implanted telemetry device to record BP beat by beat. This approach was repeated on PD 75 to test whether any effects of prenatal exposure on the stress response dissipated as the pups matured. Therefore, in this portion of the study we hypothesize that any effects of perinatal OXY exposure observed at 40 days of age would be smaller or absent when tested in adult animals (i.e., 75 days of age, when the animals are reproductively competent). We now report that there is no between-group difference in the C_1_ BP response, but that C_2_ pressor event was larger in the offspring exposed to OXY *in utero* when tested at PD 40, though not when tested at PD 75. Finally, we also report for the first time that the nature of the HR response to CS+ changes with age, and that drug exposure during gestation affects the nature of this response when tested at PD 40.

## Methods

### Animals and prenatal treatments

Virgin female Sprague-Dawley (SD) rats (Harlan, Indianapolis, IN) weighing 194–223 g were housed individually and maintained in a 14 light–10 dark photoperiod (lights on at 0500) at 22–25°C with regulated humidity. Rat chow and water were provided *ad libitum*. The study protocol was approved by the University of Kentucky Institutional Animal Care and Use Committee.

Once released from quarantine, the females were fitted with a right atrial cannula (Mactutus et al., 1994; Mactutus, 1999) with a subcutaneous, dorsally implanted access port, and allowed to recover for 1 week. During this time the cannulae were flushed daily via the subcutaneous port with sterile heparinized saline (0.4 cc, 100 IU/ml). Daily vaginal lavages were obtained to determine estrous cyclicity. In order to avoid the physiological consequences of transporting and cannulating females in early pregnancy, we bred virgin females (>200 g) with proven breeder males after cannulation surgery. Beginning 1 week after cannulation, the females were group housed with males for breeding. The day that sperm were detected via a vaginal smear was designated gestation day (GD) 0, and the females were individually housed thereafter. To minimize the physiological effects on the offspring of being raised by drug exposed mothers, additional females, destined to serve as foster mothers (see below), were also bred at the same time; these females were not cannulated or exposed to drug treatments throughout their gestation.

The pregnant rats were randomly assigned to either control (CON, normal saline vehicle) or OXY treatment groups. From GD 8 to 21, experimental dams were slowly injected over 10 min via the atrial cannula with OXY hydrochloride (2 mg/kg/day; *n* = 5 dams) (Mallinckrodt, St. Louis, MO) in normal saline solution (NSS). This dose was selected based on our pilot study that the dams were able to tolerate this dose without disturbance of litter size or birth weights of rat pups and that it is adequate to create opiate effects. We have previously reported changes in stress hormones of the offspring after exposures to intravenous 0.8 mg/kg/day of OXY during gestation (Sithisarn et al., 2008). Davis et al. had used escalating oral dose to as high as 15 mg/kg/day in their model and reported impaired spatial learning and/or memory in the offspring after prenatal OXY exposure (Davis et al., 2010). Control dams were given 1 ml/kg NSS once daily (*n* = 6 dams).

Births occurred on GD 21–22. Once delivered, all pups were counted and weighed. On PD 2, all litters were adjusted to contain 10-11 pups with equal numbers of male and female pups when possible. Since the pups' brain development in the first PDs corresponds to the third trimester of human fetal brain development, and withdrawal symptoms may affect maternal nursing behavior, the dams continued to receive OXY or NSS injection on PDs 1, 3, and 5 at the same dosage delivered during gestation. On PD5, all pups in each litter were fostered to untreated foster dams (see above). The pups were weighed daily and weaned at PD 25 when they were separated by sex. After weaning, the pups were randomly assigned to the experimental groups. For statistical analysis, data from all pups within a given litter were averaged to generate one data set per dam as described below.

### Experiment: classical conditioning

#### Subjects

Male rat pups were randomly selected on PD 27–30 from CON (*n* = 12 pups from 6 dams) and from OXY-treated litters (*n* = 11 pups from 5 dams) for the classical conditioning study.

#### Implantation of the telemetry

Arterial BP telemetry probes (PhysioTel™, Model PA-C40, Data Science International, MN) were implanted in each experimental pup at PD 27–30 days of age using standard rodent survival surgery techniques. The animals were anesthetized (sodium pentobarbital, 50 mg/kg) and the abdominal aorta exposed via a laparotomy. The sensory element of the implantable telemetry probe was placed into the aorta via puncture such that its tip pointed toward the heart (i.e., “upstream”). The body of the probe (i.e., that contains the necessary circuitry, transmitter and battery) was secured to the interior abdominal wall. The incision was closed and the skin approximated by wound clips. The animals were placed on a warm pad and were monitored until they recovered from surgery. Upon arousing they were returned to their home cage. The rats were allowed a minimum of 3 days to recover before experiments commenced.

#### Behavioral conditioning

Details of the conditioning paradigm have been published (Randall et al., 1993, 1994). Briefly, the animals were habituated to handling and restraint in a comfortable conical cloth sock for 1–2 h daily for 2 days. The animal was free to emerge from the restraint, but was immediately reintroduced to the sock until, by the end of the second day, it tended to “snuggle” at the apex of the cone with only occasional attempts to exit. Each rat was then exposed to five sets of a tone that would eventually become the “stress stimulus” and a tone that eventually would become the “non-stress stimulus”. The stressful stimulus (CS+) consisted of a 15-s pulsed tone; on the last tone of this first day of training, and on all subsequent presentations, CS+ was followed by a 0.5-s tail shock, the unconditional stimulus (US). The intensity of the shock was adjusted to the lowest level that caused the rat to flinch and vocalize (squeak); the intensity usually ranged between 0.2–0.3 mA and never exceed 0.3 mA. The 15 s, non-stressful stimulus tone (CS−) was identical to the CS+ tone except it sounded continuously (i.e., the tone was not pulsed), and was never followed by a shock. Tones were presented in random pairs (e.g., CS+, CS−; CS−, CS+ …). A minimum of 5 min elapsed between tone presentations. Training in the conditioning paradigm continued for two additional days during which 5 CS+ and 5 CS− were presented daily.

### Data acquisition and analysis

Conditioning trials were conducted starting at PD40 and, in some pups, again starting at PD75. In each case the rat was restrained in the cloth shock and an initial single day's set of 5 CS+ and 5 CS− trials was conducted to “refresh” the conditional response; the BP and HR data from these trials were not used in data analysis. Over the next 2 days additional sets of 5 CS+ and 5 CS− trials were conducted during restraint and these data were retained for subsequent analysis of the conditional CV response. Digital data sampling began 15 s before the onset of the tone and continued for 30 s (i.e., until 15 s after tone-off). Data from conditioning trials from a given rat were ensemble averaged (see below) for that pup; data from pups born of a common dam were, in turn, averaged together to yield a single data set for each OXY and each CON-treated dam. BP was digitally sampled at 500 Hz using an analog-to-digital converter (Data Translation 2810) and a microprocessor. HR was determined from the pulsatile BP signals. The programs (Vii soft, Lexington, KY) were developed for a 32 bit operating system (Windows NT) using Microsoft Visual C++ with foundation class in order to utilize large data files. The digital files of the BP recorded during 10 CS+ were ensemble averaged for each rat to yield a “high resolution” analysis of the conditional response for that individual (Randall et al., 1993, 1994); likewise for CS− trials.

**Figure 1:**
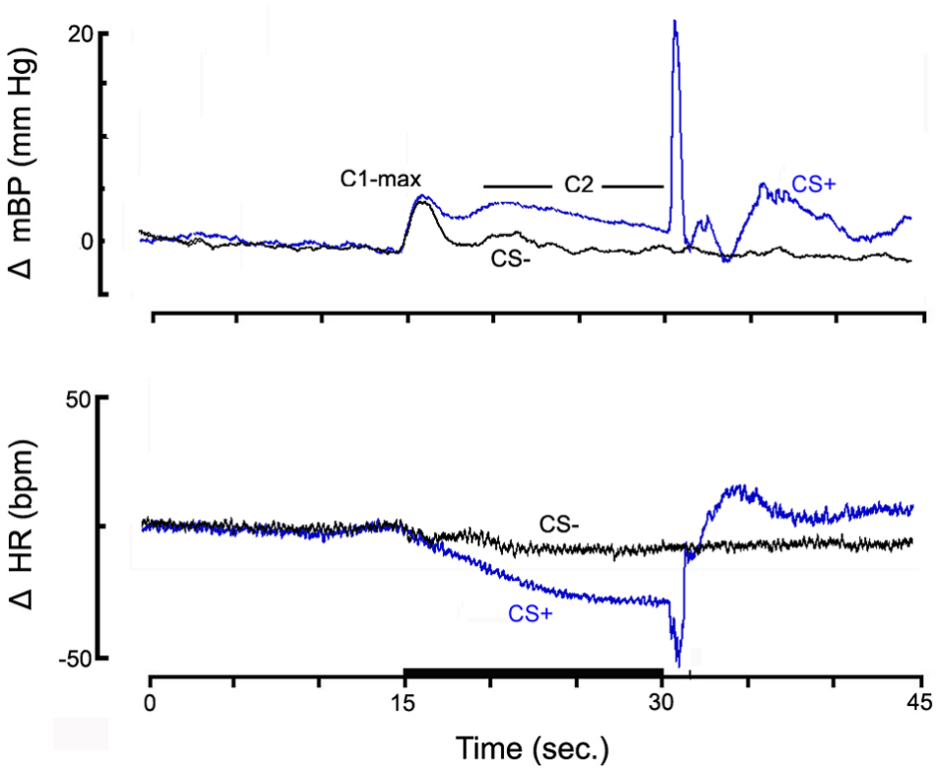
**The high resolution analyses of the change (Δ, relative to baseline) in mean arterial blood pressure (mBP; top panel) and in HR (bottom panel) for pups from control dams (*n* = 6) to CS+ tone (blue) and to CS− tone (black), on post natal day 40**.

The data analysis program quantified the conditional response from the ensemble data files. For each individual rat the mBP and HR averaged over the 15 s immediately preceding the tone was taken as the baseline, and all aspects of the response pattern were assessed as changes relative to this baseline. The initial increase in mBP was assessed as the maximum change observed within the first 2 s after the tone onset (i.e., C_1_-Max). The time when C_1_-Max occurs (i.e., t C_1_pk) was determined with respect to tone onset. C_2_-Avg was the average value of mBP during the final 10 s of the tone; this interval is indicated in Figure 1. The unconditional response (UR) is given as the maximum BP response occurring within the 3.5 s following the end of the tone. The HR corresponding in time to each of the BP values, above, was also recorded. Note that the BP data between the third and fifth seconds of the tone were discarded since they included the fall in pressure that separates C_1_ from C_2_ (Randall et al., 1993, 1994).

The data were analyzed using a linear mixed model in which the presence or absence of OXY exposure is the independent variable and the physiologic parameters (HR, mBP) are dependent variables. All findings are reported as mean ± SEM. Statistical significance was defined as *p* < 0.05.

## Results

### Parturition, litter size, and body weights

There were no differences between the OXY and CON pups in timing of parturition, litter size and body weight, either male or female, from birth to PD 32 (*p* > 0.05). For the OXY and CON male pups, mean birth weights (SEM) were 5.41(0.31) g and 4.92 (0.11) g respectively. Neither were there differences in body weights of the pregnant rats between the two groups before or after delivery.

### Baseline mean arterial blood pressure and heart rate, PD 40

Average baseline (i.e., pre-tone) mBP was lower in perinatal OXY exposed offspring (OXY: 114.8 ± 1.0) compared to pups from dams exposed to CON (118.3 ± 1.0 mmHg) (*p* = 0.02). Baseline HRs were not different between the two treatment groups (CON 462 ± 10.8 bpm; OXY: 456 ± 11.3 bpm).

### Conditional cardiovascular response, PD 40

#### Group averaged CS+ and CS− trials

Figure 1 shows the high resolution analyses of the change (Δ, relative to baseline) in mBP (top panel) and in HR (bottom panel) averaged across all pups from NSS treated dams (*n* = 6) in response to the CS+ tone (blue) and the CS− tone (black). Data are shown starting 15 s prior to tone onset and extending for 15 s after the half second shock delivery (or, for CS−, tone-off). The mBP increased to an initial peak (C1-max) immediately following tone onset for both CS+ and CS−. [Recall that the CS− tone was identical in frequency and amplitude to the CS+ tone so several tenths of a second elapsed before the animal could determine if a given tone was pulsed (CS+) or steady (CS−); hence the initial response to CS−]. The increase in mBP was sustained in response to CS+ as seen by the clear C_2_ that extended throughout the latter seconds of the trial. Conversely, mBP decreased to baseline during CS− after the initial C_1_ increase. HR modestly decreased within seconds in response to onset of both tones; it remained below baseline throughout CS+ but returned toward baseline for CS−. The UR to the tail shock for CS+ trials consisted of an increase in mBP and in HR. There were no corresponding sustained changes following the CS− tone.

**Figure 2:**
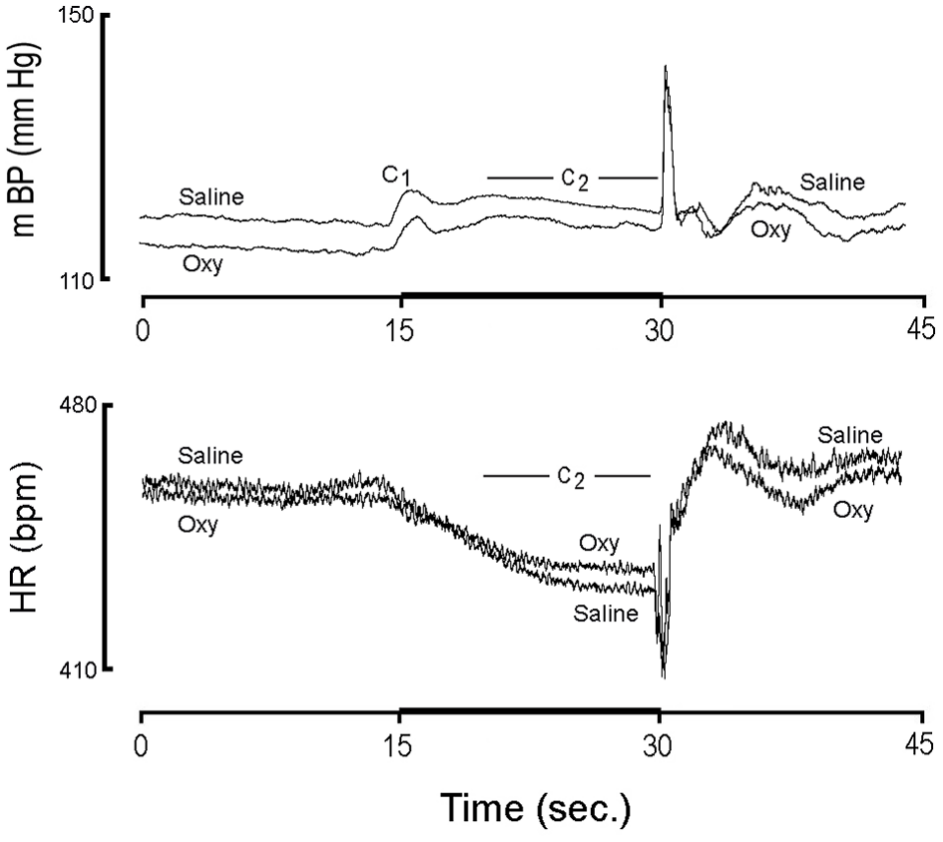
**The actual value (i.e., not normalized to baseline) for mBP and HR for conditioning trials from pups born from 6 controls (Saline) and 5 oxycodone (Oxy) dams on post natal day 40**.

Figure 2 shows the actual value (i.e., not normalized to baseline) for mBP and HR for conditioning trials from pups born from 6 CON and 5 OXY dams. The lower baseline mBP in pups from OXY dams, which was described above, is easily discerned. Likewise, the similarity in baseline HRs between the two groups is clear. The individual components of the mBP and HR responses to CS+ and CS− are presented below.

#### Mean arterial BP conditional response, PD 40

As can be discerned qualitatively in Figure 2, the initial, short-latency C_1_ peak increases in mBP after CS+ onset were not different between CON (+5.1 ± 0.4 mm Hg) and OXY pups (+5.7 ± 0.4 mm Hg). Although the magnitude of the peak change in C_1_ mBP (Δ C_1_pkBP) did not differ between CS+ and CS− tones for either group, the average value of mBP throughout the C_1_ event was significantly larger during CS+ as compared to CS− tones for both groups with no significant group x tone interaction. Finally, the time at which the peak C_1_BP (t C_1_pk) was attained relative to tone onset (i.e., evaluated for both CS+ and CS−) was similar for CON (0.74 ± 0.13 s) and OXY pups (0.84 ± 0.14 s).

**Figure 3:**
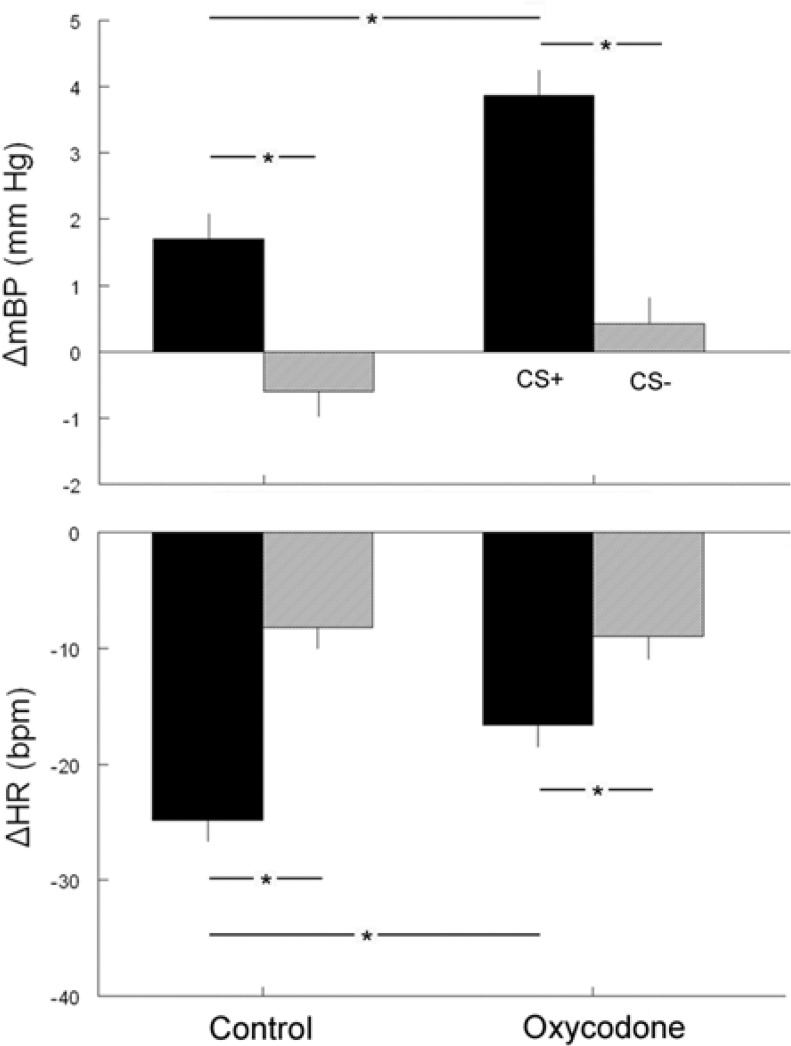
The corresponding changes from baseline in BP (top) and HR (bottom) during the conditional response between the two tones CS+ (dark bars) or CS− (gray bars) in control and oxycodone animals on post natal day 40 (^*^p < 0.05).

The second component (C_2_) of the mBP response, that is sustained throughout the last 10 s of the tone, and the corresponding change in HR (see below) are of particular interest with respect to an animal's ability to acquire the conditional response and to discriminate between the two conditions (Randall et al., 1993, 1994; El-Wazir et al., 2005). CS+ produced a larger C_2_ pressor response (Δ C_2_BP) in rats from OXY-treated dams (+3.9 ± 0.4 mm Hg) as compared to CON pups (+1.7 ± 0.4 mm Hg) (Figure 3, top). This difference persisted even when corrected statistically for differences in baseline values. Both OXY and CON rats discriminated between CS+ and CS−, as reflected in a significant difference in Δ C_2_BP between CS+ and CS− (CON CS−: −0.6 ± 0.4 mm Hg; OXY CS−: +0.4 ± 0.4 mm Hg). The group × tone interaction, however, was not significant.

There were no between group differences in any aspect of the animals' mBP response to shock delivery itself (UR BP). Likewise, there were no differences in the mBP during the 15 s following shock delivery (i.e., recovery).

#### HR conditional response, PD 40

The cardio-deceleration that occurs during CS+ concomitantly with the C_2_ pressor response, but which is not sustained during CS−, as shown in Figure 1, is another hallmark of the discrimination between CS+ and CS−. Figure 2 suggests that the slowing during CS+ is less in the OXY as compared to the CON rats. In fact, statistical analysis of actual HR values, of actual HR controlled for baseline differences, and of changes in HR during C_2_ (Δ C_2_HR) confirms that the OXY rats' bradycardia during C_2_ was smaller than in CON (Figure 3, bottom). In particular, the −24.8 ± 19 bpm slowing observed in the CON during the last 10 s of CS+ significantly exceeded the −16.6 ± 2.0 bpm observed in OXY; moreover, there was a significant group x trial interaction [*F*_(1, 21)_ = 9.37]. This difference in the change in HR persists when the effect of the somewhat different baseline HR is controlled for statistically.

### Baseline mean arterial blood pressure and heart rate, PD 75

We maintained a subset of pups from CON and OXY through an age of 75 days post-delivery to determine if any between group differences were accentuated or diminished with age. Mean baseline BPs in adults were higher than those on PD 40, but the overall baseline BPs in OXY, were not significantly different from CON (OXY: 143.4 ± 1.7 vs. CON: 149.1 ± 2.8 mmHg; *p* = 0.1). Baseline HRs were remarkably lower at PD 75 than at PD 40 for both CON (396 ± 21 bpm) and for OXY (395 ± 13 bpm), but, again, there were no between group differences.

### Conditional cardiovascular response, PD 75

#### Mean arterial BP conditional response, PD 75

After adjustment for the baseline BP, C_1_pk BP, Δ C_1_pk BP, C_1_avg BP, and Δ C_1_BP were not different between OXY and CON rats, either during CS+ or CS−. Time to the peak C_1_ mBP increase was not different between OXY and CON either during CS+ or CS−.

The significant difference in the amplitude of the Δ C_2_BP response during CS+ observed at PD40 disappeared by PD 75 (CON: +3.4 ± 2.3 mm Hg; OXY: +4.5 ± 1.4 mm Hg).

Importantly, CON rats were able to differentiate between CS+ and CS−, as demonstrated by an increased C_2_BP during CS+ but not for CS− (CS+: 155.4 ± 2.7 vs. CS−: 147.8 ± 2.7 mmHg; *p* = 0.02). Conversely, even though OXY rats could differentiate between CS+ and CS− at younger age, they did not retain this ability during adulthood (CS+: 147.1 ± 1.6 vs. CS−: 145.9 ± 1.6 mmHg, *p* = 0.49). These discrepancies persisted after controlling for the baseline values or when comparing using Δ C_2_pk BPs (CON CS+ vs. CS−: *p* = 0.029, OXY CS+ vs. CS−: *p* = 0.14).

#### HR conditional response, PD 75

A major difference in the conditional HR response at PD 40 was that the CON animals slowed rate more during C_2_ than did the OXY animals. At PD 75 there were no between group differences in Δ C_2_HR during CS+, and, in fact, the conditional bradycardia at PD 40 was no longer elicited during C_2_ at PD 75 (CON: −2 ± 17 bpm; OXY +2 ± 10 bpm).

UR HRs were similar between CON and OXY rats, during both CS+ and CS−. Finally, there were no between group differences in recovery mBP or HR.

## Discussion

### PD 40

This study has demonstrated quantitative differences in baseline mBP and in select aspects of the CV response to an acute behavioral stress in rat pups born of dams exposed during gestation to OXY as opposed to control pups born of dams exposed to saline. The conditional response is advantageous for a study such as this because a great deal is known about the underlying mediation of the changes in mBP and in HR, and because the response pattern is reproducible and stable over time. Moreover, the response pattern can be elicited multiple times at the investigator's discretion. Major findings are that at 40 days of age rat offspring in the OXY group as compared to the CON had a modestly, but significantly, lower baseline mBP (with no difference in baseline HR), and a larger increase in mBP during the C_2_ component of the conditional response with a concomitantly smaller decrease in HR. There was no between group difference in the C_1_ component of the BP conditional response. These findings can be interpreted in terms of what is known about the mediation and control of the conditional CV response pattern in the mature SD rat.

The short-latency conditional increase in mBP, which we call C_1_, is preceded by a large-amplitude, but short-lived “sudden burst” (SB) in sympathetic nerve activity (SNA) in SD rats (Randall et al., 1994); the amplitude of the SB correlates with the amplitude of the C_1_ pressor response (Burgess et al., 1997). The C_1_ BP increase is produced by an increase in peripheral resistance; in fact, there is little or no concomitant change in either stroke volume or HR and, thereby, none in cardiac output (Li et al., 1998). As noted previously, C_1_ originates as an orienting or startle response (though it subsequently attains properties of a conditional response); that is, no “learning” is initially required for the animal to demonstrate this component of the response (El-Wazir et al., 2005). It is noteworthy, therefore, that there were no between group differences in the present study in any aspect of C_1_, including its latency with respect to tone onset. That is, OXY exposure *in utero* did not affect this “intrinsic” aspect of an acute stress response.

The C_2_ pressor event, which occurs following the SB in SNA, is accompanied in time by a moderate (*ca.* +24%), but sustained increase in sympathetic activity (Randall et al., 1994). Relative to baseline, cardiac output increases during C_2_ by 2 ± 1 ml/min while peripheral resistance decreases on the average by 4 ± 2 dyn s/cm^5^ in the SD strain (Li et al., 1998). The sustained C_2_ mBP increase is dependent, therefore, upon the heart's developing and maintaining an increase in cardiac output over baseline. In contrast to C_1_, C_2_ is acquired as the animal learns the association between the CS+ tone and the US shock (El-Wazir et al., 2005)—the rat must learn the tone/shock pairing to display a C_2_. It is again particularly noteworthy, therefore, that the C_2_ mBP increase was significantly larger in the OXY animals than their controls. This implies that the drug exposure *in utero* impacted “higher” cognitive function with effects that can be detected in the offspring's learned response pattern.

The nature of the C_2_ HR change during CS+, if any, is species-dependent (compare Randall et al., 1994; Li et al., 1997, 1998; Brown et al., 1999). To date we had studied only adult rats and HR is essentially unchanged (Randall et al., 1994) or decreases by only ~5 bpm (Li et al., 1998) relative to baseline in adult SD rats during the last 10 s of CS+. The CS+ C_2_ bradycardia is eliminated by atropine in Zucker lean and obese rats, but only modestly (though significantly) attenuated by beta-adrenergic blockade (El-Wazir et al., 2008). The HR slowing is therefore attributable primarily to elevated parasympathetic nervous drive to the SA-node, probably via the baroreflex secondary to any C_2_ mBP increase. In the context of these previous studies two current observations are remarkable. First, in the young SD rats of both groups, in contrast to the SD adult, HR significantly decreased during C_2_ relative to baseline (Figure 1). Second, the C_2_ HR decrease was significantly smaller in the OXY vs. CON group, despite the larger C_2_ mBP increase in the OXY vs. CON. This latter observation implies either that the parasympathetic control of HR is somewhat impaired in the OXY animals, or that the “gain” of their baroreflex is smaller than the controls, or perhaps both conditions obtain.

A clear difference in the nature of the CV response to CS− vs. CS+ is indicative of the subject's ability to discriminate between the two behavioral situations. In the conditioning paradigm, discrimination such as this demonstrates that the response pattern is truly a learned behavior, and not simply an erratic response to any given event (Randall et al., 1993). The ability of the SD rat to demonstrate such discrimination is acquired over successive trials during the “acquisition” phase of training—as the animal learns, or acquires the conditional response (El-Wazir et al., 2005). Each group clearly demonstrated the ability to discriminate CS+ from CS−, both by the relatively smaller C_2_ mBP increase and smaller HR decrease during CS−. In other words, prenatal exposure to OXY did not demonstrably impair this aspect of the OXY animals' ability to learn the behavioral paradigm at PD 40.

### PD 75

As animals in both groups matured baseline mBP rose and baseline HR fell; the significant difference observed at PD 40 in baseline mBP disappeared. Moreover, the significant difference in Δ C_2_BP at PD 40 also disappeared. These findings indicate that, as the OXY rats matured, the effects of their prenatal exposure to OXY upon their response to the acute stress dissipated. Finally, the significant HR slowing during C_2_, which is not characteristic of the (adult) SD, was no longer evoked during CS+ at PD 75, indicating that the nature of the conditional HR response changes with maturation.

The baseline mBPs in both groups at PD 75 (i.e., CON 149 mm Hg, OXY 143 mm Hg) were higher than we expected. That stated, the 75 day old animal is younger than animals in which we have typically recorded pressure, so it may be that at this earlier developmental stage the mBP is higher than we observe in the mature rat. In fact, Litchfield reported a progressive increase in mBP from birth to PD 35 (*mBP* = 109.6) in anesthetized rat pups, but he did not follow their pressures further and the trajectory in the rise of mBP appeared to be leveling by PD 35 (Litchfield, 1958). Kasparov and Paton (1997) also reported an upward progression in anesthetized rat pups from PD 6 to 45 (*mBP* = 74.6 mm Hg), but with no additional statistically significant increase at PD 45 (Kasparov and Paton, 1997). We reported beat-by-beat mBP via telemetry averaged over 24 h in rats ~60–90 days of age while in their home cages to be ~98 mm Hg, and that mBP gradually declined thereafter as the animals matured (Anigbogu et al., 2012). By comparison, we reported (Hoyt et al., 2013) a *mBP* of 127.6 ± 13.5 (SD) mm Hg via catheter in behaviorally conditioned adult rats during the 15 s baseline (i.e., as in the present study), which is clearly higher than our value from the 24 h telemetry. The present pups were not subject to the sock restraint or periodic handling between measurements at PD 40 and at PD 75, so the unexpectedly high mBP perhaps is attributable to the relatively unaccustomed restraint on PD 75.

### Prenatal opiates effects on the autonomic nervous system

To date there are no human or animal studies that directly explore the effects of prenatal OXY on BP and autonomic system controls; however, there is evidence both from human and animal studies suggesting that the autonomic nervous system is affected by the exposure to opiates *in utero*. Many human neonates prenatally-exposed to opiates experience symptoms of the neonatal abstinence syndrome which are autonomic regulated functions (e.g., increased sweating, nasal stuffiness, fever, mottling, and temperature instability) (American Academy of Pediatrics, 1998; Bandstra et al., 2010). To study autonomic control in children, vagal tone adaptation, among other methods, has been used as an indicator of autonomic regulation in the setting of prenatal cocaine exposure (Sheinkopf et al., 2007). The variability in HR that occurs at the frequency of breathing, or respiratory sinus arrhythmia (RSA), reflects the parasympathetic influence on HR variability via the vagus nerve (Randall et al., 1991; Berntson et al., 1993; Calkins and Keane, 2004; Yasuma and Hayano, 2004). Suppression of RSA seen on electrocardiography has been considered an adaptive response indicative of removal of the vagal brake to increase metabolic output in order to engage more effectively with the environment (Porges, 1995, 2007). In general, higher levels of baseline parasympathetic activity as measured by RSA and/or the ability to suppress parasympathetic activity are related to enhanced autonomic emotional regulation and its developmental outcomes (Calkins and Keane, 2004; Stifter et al., 2011). RSA suppression during an attention demanding task was impaired in school-aged boys who were exposed to opiates (heroine/methadone) *in utero*, suggesting possible long term effects of opiates on the (dis)organization of the vagal system (Hickey et al., 1995). However, this finding was inconsistent with a subsequent study which showed that when an extrinsic incentive, and tasks that were interesting, were offered, RSA suppression in opiate-exposed school-age boys was comparable to the controls (Suess et al., 1997).

Animal studies have shown that prenatal opiates induced changes in sympathoadrenal activity, although the direct effects of these changes on BP and HR have not been previously examined. For example, under resting conditions, adult male rats prenatally exposed to morphine had decreased adrenal noradrenaline (NA) and adrenaline contents, but increased circulating levels of adrenaline (Dutriez-Casteloot et al., 1999). Under ether inhalation stress, these rats had hypo-responsive SAM activity; adrenal norepinephrine was decreased at 90 min after inhalation and the compensatory biosynthesis of adrenal catecholamines did not adapt appropriately to stress when compared to controls (Laborie et al., 2005).

The possible underlying mechanisms of changes in autonomic control after prenatal exposure to OXY remain to be investigated. The enhanced C_2_ mBP increase in the OXY animals implies either that they have a larger increase in SNA evoked by the acute stress, or that the effector response (i.e., vascular smooth muscle and/or myocardium) to a given increase in SNA was enhanced in OXY animals. Changes in the regulatory functions of κ-ORs on the myocardium may also contribute to the enhanced mBP increase. OXY acts, besides on μ-OR, on κ-OR (Ross and Smith, 1997). The κ-opioid system works closely with the sympathetic nervous system in the regulatory functions of the heart (Wong and Shan, 2001). Endogenous κ-opioid peptides (dynorphins) are found in the sympathetic nerve fibers and ganglion cells (Steele et al., 1996). Chemical sympathectomy reduces the amount of dynorphin in the heart, indicating that κ-opioid peptides may co-exist with the catecholamines in the sympathetic nerve terminal (Wegener and Kummer, 1994; Pepe et al., 2004). The activation of κ-OR with a selective exogenous agonist U50, 488H inhibits the effects of β-adrenergic receptor (β-AR) agonist to increase rat myocyte contractility (Yu et al., 1998). These inhibitory effects are antagonized by a selective κ-OR antagonist, indicating that the effects are κ-OR mediated (Yu et al., 1998). A disturbed cross-talk between κ-OR and β-AR (Pepe et al., 2004) by significant reduction in or absence of the inhibition of β-AR stimulation by κ-OR stimulation may lead to an excessive increase in cardiac activity leading to disproportionately increased BP (Wong and Shan, 2001). Chronic exposure to other opioid agonists such as morphine causes receptor internalization, and changes in receptor binding or post-translational modification and receptor biosynthesis (Patel et al., 2002; Przewlocki, 2004; Nagi and Pineyro, 2011). Thus, one can speculate that long term *in-utero* exposure to a κ-OR agonist such as OXY may down-regulate the expression of κ-OR in cardiac myocytes and in turn, reduce the inhibition of β-AR stimulation during stress and lead to significantly increased C_2_ mBP in the OXY animals.

### The effects of prenatal opiates on learning/memory and cognition

To date there have been very few human studies that identify the effects of prenatal opiate exposure on cognitive development and learning, and most of those which have been published were conducted in children born to heroine or methadone dependent mothers who also used other illicit drugs. Thus the outcomes were confounded by the effects of other drugs and psychosocial factors. Hyperactivity, lack of concentration and aggression were reported in these children (Olofsson et al., 1983). Cognitive deficits in opiate-exposed children were noted at various ages in a few studies (Van Baar and De Graaff, 1994; Pulsifer et al., 2004; Steinhausen et al., 2007). Previous reports indicate that exposure to other opiates prenatally is associated with impaired learning/memory (Niu et al., 2009; Wang and Han, 2009; He et al., 2010). More recently, Davis et al. (2010) used an animal model to study the effect of prenatal oral OXY exposure on learning and/or memory in adult male rats. OXY rats showed a decreased use of spatial strategies and increase in non-spatial strategies in the Morris water maze. Interestingly, OXY rats had a modest but significant retention deficit in T-maze tasks when assessed 5 days after acquisition training ended (Davis et al., 2010). This is consistent with our findings that even though OXY rats were able to learn to differentiate between CS+ and CS− at age PD 40, they were not able to retain this ability when tested at PD 75, even if the procedure was repeated prior to the test with set of 5 CS+ and 5 CS− trials to “refresh” the conditional response at this age. These findings suggest that perinatal OXY exposure may be associated with impairment of formation and/or storage of memory. The mechanisms for this memory deficit remain to be elucidated but there is evidence that prenatal exposure to other opiates is associated with alterations in hippocampal cholinergic function (Vatury et al., 2004), glutamatergic neurotransmission (Tao et al., 2001; Yang et al., 2003), hippocampal synaptic complex (Lin et al., 2009) and increased hippocampal neuronal apoptosis (Wang and Han, 2009) which may lead to memory/cognitive deficits.

## Conclusions

In conclusion, perinatal OXY exposure is associated with an increased BP response to the “learned” component of an acute behavioral stress in the young adolescent male rats, suggesting increased SNA input or increased response of the effectors. This difference dissipated when the stress was repeated as the rats matured to adult age. Adult prenatally-OXY exposed rats also had an impaired retention of the learning of this conditioning at younger age, which may result from a memory deficit associated with prenatal opiate exposure.

### Conflict of interest statement

The authors declare that the research was conducted in the absence of any commercial or financial relationships that could be construed as a potential conflict of interest.
